# Characteristics of discrimination and ambulatory cognitive performance among older Black and White adults

**DOI:** 10.1007/s12144-024-07266-w

**Published:** 2025-02-08

**Authors:** Erin E. Harrington, Alyssa A. Gamaldo, Martin J. Sliwinski, Jonathan G. Hakun, Orfeu M. Buxton, Mindy J. Katz, Carol A. Derby, Kaylee Foor, Christopher G. Engeland, Jennifer E. Graham-Engeland

**Affiliations:** 1 Department of Biobehavioral Health, The Pennsylvania State University, University Park, PA, USA; 2 Center for Healthy Aging, The Pennsylvania State University, University Park, PA, USA; 3 Department of Psychology, University of Wyoming, Laramie, WY, USA; 4 Department of Psychology, Clemson University, Clemson, SC, USA; 5 Department of Human Development and Family Studies, The Pennsylvania State University, University Park, PA, USA; 6 Department of Neurology, College of Medicine, The Pennsylvania State University, Hershey, PA, USA; 7 Department of Neurology, Albert Einstein College of Medicine, Bronx, NY, USA; 8 Ross and Carol Nese College of Nursing, The Pennsylvania State University, University Park, PA, USA

**Keywords:** Minority and diverse populations, Discrimination, Ambulatory cognition, Ecological momentary assessment, Older adults

## Abstract

Perceived discrimination has been linked with neurocognitive disparities between Black and White adults. Yet, cognitive assessments outside of laboratory settings and the relevance of perceived reasons for discrimination require additional attention. The present work addressed associations between discrimination and ambulatory cognitive performance (i.e., spatial working memory, short-term memory binding, processing speed) in ecological settings among older Black and White adults enrolled in the Einstein Aging Study. Consistent with past laboratory-based research, Black adults exhibited worse ambulatory cognitive performance and reported more frequent discrimination compared to White adults. Racially stratified analyses examined characteristics (i.e., number and type) of the perceived reasons for discrimination as moderators in relation to discrimination frequency and cognition. For Black adults who endorsed zero or one reason for discrimination compared to those who endorsed multiple reasons, discrimination frequency was associated with worse spatial working memory. Additionally, among Black participants who did not attribute discrimination to their race compared to those who endorsed racial discrimination, discrimination frequency related to worse spatial working memory. For White adults, cognitive performance was largely unrelated to discrimination frequency and characteristics. Findings highlight the value of examining discrimination and cognition in daily life, and the importance of assessing characteristics of discriminatory experiences within racial groups.

## Introduction

Discrimination is a pervasive social stressor with implications for older adults’ cognitive health (Shankar & Hinds, 2017; Zahodne et al., 2017, 2019). The links between discrimination and cognition within the extant literature are well documented. However, much of this work has examined cognition using single-assessment neurocognitive batteries often collected within laboratory or diagnostic settings (e.g., Barnes et al., 2012; Keating et al., 2021; Meza et al., 2022); what is missing from the literature is an examination of discrimination and cognitive performance within everyday settings. Furthermore, the field would benefit from addressing everyday cognition in association with characteristics of discriminatory experiences beyond the frequency of events, such as the reasons perceived to underly discriminatory experiences (i.e., specific type of discrimination, endorsed types of discrimination count). Assessing such characteristics of discrimination may help contextualize the impact of discrimination frequency on cognition by identifying whether specific types of discriminatory experiences are more impactful for certain individuals (e.g., Sutin et al., 2015) and whether experiencing multiple forms of discrimination (Cobb et al., 2022; Gayman & Barragan, 2013; Grollman, 2012), compounds the negative effect of discrimination on cognition. Thus, the current study investigates the interactive effects between discrimination frequency and characteristics on cognitive performance within daily settings among older Black and White adults.

## Discrimination and cognition

Discrimination is an important factor that contributes to cognitive health in late life (Shankar & Hinds, 2017; Zahodne et al., 2017, 2019). For example, past research has found there to be domain-specific impacts of discrimination on older adults’ cognitive abilities when examined within laboratory or diagnostic settings. One study by Zahodne et al. (2020), that utilized a battery of neurocognitive assessments to measure performance across five cognitive domains, found a negative association between discrimination and both executive functioning and visuoconstruction after accounting for depressive symptoms and vascular disease. In other related work, discrimination was linked with memory recall, but not with verbal fluency, after controlling for various demographic variables (Shankar & Hinds, 2017). There is also evidence of a longitudinal link between discrimination and cognitive performance (Zahodne et al., 2019), with some work indicating that perceived everyday discrimination relates to poor episodic memory performance both concurrently and six years later (Zahodne et al., 2017).

While discrimination is prevalent among many older adults (e.g., Ayalon & Gum, 2011; Rippon et al., 2014; Shankar & Hinds, 2017), it should be noted that individuals identifying with certain demographic groups are more likely to experience discrimination. Specifically, work by Ayalon and Gum (2011) indicates that older Black adults experience significantly higher levels of everyday discrimination compared to other racial groups, including White adults. It is further likely that differential experiences of discrimination may uniquely contribute to Black adults’ cognition in older adulthood. Several studies support this logic, as more frequent perceived discrimination has been related to poor cognitive abilities among older Black Americans, including worse episodic memory and perceptual speed (Barnes et al., 2012). Additionally, discrimination appears to have a lasting influence on memory, language, visuospatial abilities, and processing speed (Shankar & Hinds, 2017; Zahodne et al., 2020), and may contribute to risk for Alzheimer’s disease and related dementias (ADRD) among Black adults compared to White adults (see Alzheimer’s Association, 2023).

Yet, some of the findings in this area are mixed. For instance, Meza et al. (2022) did not observe a relationship between a diverse sample of older adults’ everyday discriminatory experiences and cognition measured using a battery of cognitive tests; however, a greater extent of lifetime discrimination was associated with better cognitive performance among Black adults. Similar findings were observed by Sutin et al. (2015), such that greater perceived racism was associated with better cognitive functioning among Black adults. Given the disparate findings within the literature, further examination of discrimination and cognition is needed to better contextualize how discrimination impacts older adults’ cognition.

It is important to note that much of the work linking discrimination and cognitive performance has relied on single assessments of cognition or neurocognitive batteries (e.g., Barnes et al., 2005, 2012; Sutin et al., 2015; Williams et al., 2019; Zahodne et al., 2020) in which older Black adults tend to perform worse than older White adults (Babulal et al., 2019; Barnes et al., 2005). Studies such as these, which use cognitive assessments administrated within laboratory or diagnostic settings, provide a glimpse into cognitive performance but may not fully capture older adults’ daily cognitive abilities in ecologically valid contexts. To improve our understanding of the impact of discrimination on cognition, research is needed to address cognitive performance in everyday settings.

Technological advancements, such as ecological momentary assessments (EMA), facilitate multiple daily measurements of cognitive functioning that capture performance within daily settings, otherwise known as *ambulatory* cognitive performance. By collecting performance multiple times per day across several days, researchers can improve measurement precision in comparison to traditional single assessments of cognitive performance examined within laboratory or clinical settings (e.g., Shiffman et al., 2008; Spooner & Pachana, 2006). Importantly, ambulatory cognitive assessments exhibit strong convergent validity with traditional laboratory-based assessments (Singh et al., 2023; Sliwinski et al., 2018) while also offering improved ecological validity. Specifically, ambulatory cognitive tests capture cognition within ecologically valid settings, or settings in which we’d expect individuals to utilize their abilities. Within the context of discrimination research, examining discrimination in association with ambulatory cognitive performance will provide insight into how perceived discrimination relates to cognition in everyday life.

## Characteristics of discriminatory experiences

Research on the link between discrimination and cognition often focuses on the *frequency* of discriminatory experiences. Across several studies, older individuals who report experiencing more frequent discrimination typically exhibit poorer cognitive functioning (e.g., Barnes et al., 2012; Zahodne et al., 2017, 2020). It is important to acknowledge, however, that discrimination comes in many forms (e.g., racism, sexism, ageism). Moreover, the impact of the characteristics of discriminatory experiences (e.g., type or perceived reason for discrimination, number of perceived reasons) may differentially relate to older adults’ health outcomes (e.g., Sutin et al., 2015; Williams et al., 2019). In support of this notion, Sutin et al. (2015) examined how eight types of discriminatory experiences uniquely related to health and observed that only four (i.e., age, weight, physical disability, appearance) were associated with poor physical and emotional health. In recent work, Ferraro and Zaborenko (2023) found that Black adults exhibited better cognitive functioning compared to White and Hispanic adults, in relation to *racial* but not overall discrimination. As such, additional research is needed to better understand the characteristics of older adults’ discriminatory experiences and how they may relate to cognitive performance in daily life. To this end, the present work focuses on how the most commonly endorsed reason and the count of endorsed reasons (zero, one, or multiple) for discrimination modify the relationship between discrimination frequency and cognitive performance within daily settings among older Black and White adults.

Within the literature, many studies have focused on how specific types of discriminatory experiences relate to health outcomes. For example, Brondolo et al. (2008) observed that greater perceived racism was associated with greater daily negative affect among Black adults. Sutin et al. (2014) found that greater perceived weight discrimination was associated with increased inflammation. Pertinent to cognition, activation of age-based stereotypes has been associated with poor cognitive test performance among older adults (Lamont et al., 2015), and more frequent racial discrimination has been related to poor cognitive performance among Black adults (Barnes et al., 2012; Keating et al., 2021; Shankar & Hinds, 2017; Zahodne et al., 2020). Yet, the question remains whether these effects are apparent when examining cognition within daily settings, outside of traditional laboratory or diagnostic testing. Furthermore, it is possible that the specific type of discrimination experienced may uniquely contribute to the influence of discrimination on different cognitive outcomes. That is, more frequent perceived discrimination may relate negatively to older adults’ cognitive performance, but the magnitude of this effect may be stronger when accounting for the specific type of discriminatory experience reported by a given group of individuals.

It should be noted that some individuals experience one primary form of discrimination, whereas others experience multiple types of discrimination simultaneously. Intersec- tionality theory suggests that individuals often experience discrimination based upon a combination of marginalized social identities (e.g., race, age, gender; Harnois, 2014), which supports the observed intersectionality of discriminatory experiences among older adults of color (e.g., Black/ African Americans; Meyer, 2003). Discrimination based on intersectional identities has been theorized to facilitate the unique stressful experiences and health consequences (e.g., hypertension, diabetes, cognitive impairment) often encountered by Black adults, according to minority stress theory (Forrester et al., 2019; Mouzon et al., 2020). As such, the count (zero, one, or multiple) of the reasons perceived to underly experienced discrimination, in combination with discrimination frequency, may be particularly relevant to health outcomes among individuals with intersectional identities.

In support of this notion, research has shown significant relationships between reporting multiple sources of discrimination and poor health-related outcomes, such as greater risk for mental health conditions, compared to those who report few sources (e.g., Gayman & Barragan, 2013; Groll- man, 2012). Cobb et al. (2022) observed that among older Black adults who endorsed multiple reasons for perceived discriminatory experiences, there was a heightened risk for all-cause mortality. Although links with cognition were not explored, Cobb and colleagues emphasized that future research should examine specific combinations of reasons for discrimination as some combinations may be more detrimental to health than other combinations (see also, Sutin et al., 2015). Thus, additional research examining how the number of endorsed reasons relate to the link between discrimination frequency and cognition may help contextualize the impact of discrimination among individuals with intersectional identities.

## Current study

The present work examined characteristics of Black and White adults’ perceived discrimination and ambulatory cognitive performance. Such research is needed, as minimal studies have examined links between discrimination and cognitive performance within daily settings. We first examined demographic differences between Black and White adults’ reported frequency of discrimination and performance on ambulatory cognitive tests. Similar to previous assessments of racial differences in health-related outcomes, we hypothesized that Black adults would report more frequent discrimination (e.g., Ayalon & Gum, 2011) and would exhibit poorer performance on the cognitive tests compared to White adults (Babulal et al., 2019; Barnes et al., 2005).

Leveraging similar approaches to past literature (Sutin et al., 2015) and recent recommendations to address multiple forms of discrimination (Cobb et al., 2022), we next examined characteristics of the discriminatory experiences reported by older Black and White adults. Specifically, we examined the most endorsed type of reason (e.g., race, age, gender) and the reason count (i.e., zero, one, or multiple) underlying discriminatory experiences. We expected to see Black and White adults report different reasons for their discriminatory experiences. However, we did not have specific a priori hypotheses about which types or count of types of discriminatory experiences Black or White adults would report. Instead, we took a data-driven approach to identify each racial group’s most reported reason for discrimination. We did, however, expect that Black adults would be more likely to report experiencing multiple sources of discrimination compared to White adults, given that Black adults often identify multiple reasons for their discriminatory experiences (Cobb et al., 2022).

**Fig. 1:**
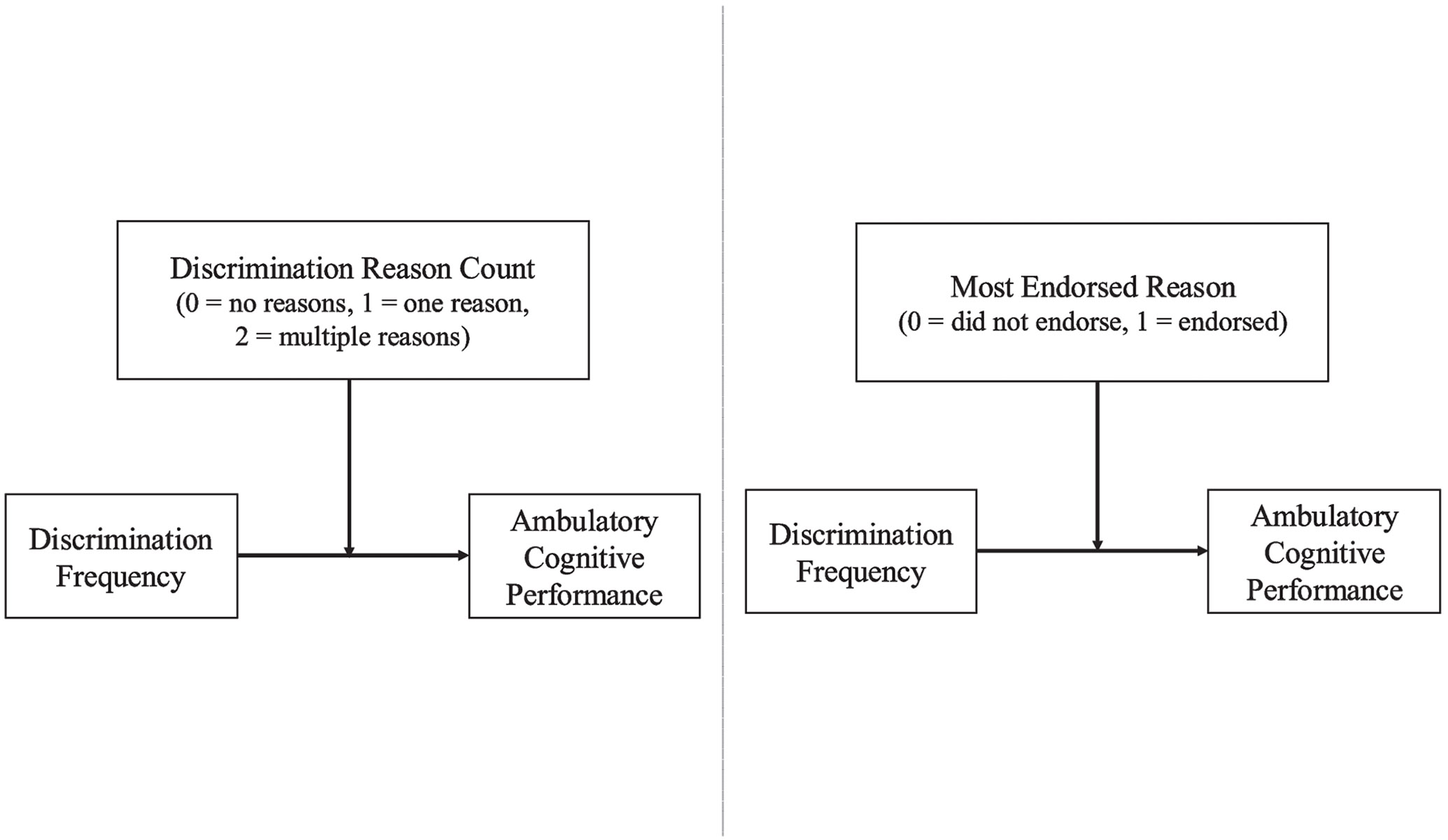
Tested models examining (1) Discrimination reason count and (2) Most endorsed reason as respective moderators in the link between discrimination frequency and ambulatory cognitive performance. Note. Analyses were stratified by race; thus models were separately tested within Black and White adults

Importantly, we utilized within-racial group analyses to explore, separately in Black and White adults, whether the characteristics of reasons perceived to underlie discrimination moderated the association between discrimination frequency and ambulatory cognitive performance (see Fig. 1). We adopted a racially stratified approach based on recent recommendations that cross-racial comparisons may not always be best practice; such studies tend to focus only on racial differences without addressing meaningful relationships that may be 1) present *within* racial groups, and 2) obscured in cross-racial designs (Baker & Gamaldo, 2022; Whitfield et al., 2008). Hence, race-stratified models enabled us to better test our hypothesis that the characteristics of Black and White adults’ perceived reasons for discrimination would moderate the link between discrimination frequency and cognitive performance in daily settings.

Consistent with past work documenting the negative effects of specific types of discrimination on cognition (e.g., Barnes et al., 2012; Zahodne et al., 2020), we hypothesized that the most commonly endorsed reason would moderate the link between discrimination frequency and ambulatory cognition. Specifically, we expected that participants who endorsed experiencing the most common reason would exhibit worse cognitive performance in association with greater discrimination frequency. Similarly, we hypothesized that discrimination reason count (i.e., zero, one, multiple) would significantly moderate the link between discrimination frequency and ambulatory cognition. As individuals with intersectional identities can be at greater risk for experiencing multiple forms of discrimination (e.g., Cobb et al., 2022), we anticipated that older adults who endorsed multiple reasons would exhibit worse cognition in association with more frequent discrimination when compared to individuals who endorsed fewer reasons to underly their discriminatory experiences.

## Method

### Participants and procedure

The current sample was drawn from the ongoing Einstein Aging Study (EAS). Participants were recruited via systematic random sampling from the New York City Registered Voter list for Bronx County, NY. Potential participants were mailed introductory letters and follow-up phone screens were conducted to determine eligibility (i.e., English-speaking, community-residing, aged 70-years or older, dementia- free; see Zhaoyang et al., 2021 for additional details). After a phone screening, eligible participants attended a research clinic visit during which they provided informed consent in accordance with study protocols approved by the local institutional review board. Participants also completed questionnaires and received training on how to use study smartphones for the EMA testing protocol. EMA testing included two practice days followed by 14 consecutive days of six testing sessions per day: self-initiated sessions at wake-up and end- of-day, and four quasi-randomly prompted sessions during participant’s self-reported waking hours.

Between May 2017 and February 2020, 308 participants completed an initial EMA protocol. The present study includes a sample of 273 EAS participants (182 women; *M_age_* = 77.2 years, *SD* = 4.9 years) that identified as non-Hispanic White (*n* = 139) or Black (*n* = 134; included “Black/African American” [*n* = 125] and “Hispanic – Black” [*n* = 9]). White participants were more educated (*t*(271) = 5.09, *p* < 0.001) and reported fewer health conditions than Black participants (*t*(271) = −2.03, *p* = 0.043) in the current study. Table 1 provides additional sample demographics.

### Measures

#### Perceived discrimination

An abbreviated version of the Everyday Discrimination scale (Williams et al., 1997) measured perceived discrimination (see Appendix in Supplementary Materials). Prior to EMA testing, participants completed eight items on the frequency (1 = *never*, 4 = *often*) of their day-to-day discriminatory experiences. Overall, the measure was reliable (α = 0.87). Responses were summed such that higher scores indicated more frequent discrimination. If participants reported experiencing discrimination, they completed the second portion of this measure that included a list of 16 possible reasons for discrimination (e.g., due to race/ethnicity, gender, age, education). Participants indicated (*Yes/No*) whether they thought each reason was an explanation for their perceived discrimination. Responses from this portion of the measure were used to examine characteristics (i.e., count and type) of reasons perceived to underlie discrimination.

#### Ambulatory cognitive tests

Ambulatory tests of cognition were administered via study smartphones during the EMA testing protocol. Performance was averaged across all sessions in the 14-day measurement period to provide an overall assessment of participants’ cognitive functioning within daily settings. Specifically, cognitive performance was assessed for the following three domains:

##### Spatial working memory

Participants completed two trials of the *grid memory* test (Sliwinski et al., 2018) per session. During each trial, participants were presented with a 5 × 5 study grid and were asked to remember the location of three red dots that appeared in three locations in the grid. After an eight second distraction task (letter cancellation task), participants were asked to recall the original location of the three dots. Performance was determined by summing the Euclidean distances between the original dot locations and the participants’ response across the two trials per session. Higher scores on this measure indicated more spatial working memory errors (i.e., poorer performance).

##### Short-term memory binding

Participants completed up to eight trials of the *color shape binding* test (Parra et al., 2010) per session. In this task, three colored shapes appeared on the screen for 2000 ms and participants were asked to remember the presented combination of shapes and colors. After a brief retention period (900 ms), the same shapes appeared in new locations and participants were asked to judge whether the colors associated with each shape matched the study set. Performance was determined based on the adjusted proportion of correct responses (i.e., hit rate minus false alarm rate), with higher scores indicating better short-term memory binding performance.

##### Processing speed

Participants completed up to 11 trials of the *symbol search* task (Sliwinski et al., 2018) per session. During each trial, participants viewed a split screen with three symbol pairs presented at the top and two symbol pairs presented at the bottom. Participants were to decide as quickly as possible which of the bottom symbol pairs matched those at the top of the screen. Performance was determined by mean response times in milliseconds, such that higher mean response times were indicative of slower processing speeds.

### Analytical procedures

Analyses were conducted using IBM/SPSS Statistics 27.0. The statistical significance threshold was set to α = 0.05. Independent samples *t*-tests examined differences in ambulatory cognitive test performance and reported discrimination frequency between Black and White adults. We were also interested in characteristics of the reasons perceived to underlie discrimination. Only individuals who experienced discrimination completed follow-up items on underlying reasons; therefore, all remaining analyses were constrained to Black (*n* = 117) and White (*n* = 98) adults who reported discrimination and therefore received questions on the perceived reasons for these experiences. Chi-square analyses examined racial differences in the count and types of perceived reasons for discrimination.

Keeping with recent recommendations, racially stratified moderation analyses (Model 1) examined participants’ number of endorsed reasons as a moderator of the association between discrimination frequency (*X*) and each cognitive outcome (*Y*s) using the PROCESS macro (Hayes, 2018). Similar to past work (e.g., Gayman & Barragan, 2013), a number of participants in the current study (*n* = 29) reported experiencing discrimination but did not provide information on the perceived reasons for these experiences. Rather than excluding these individuals or treating them as “missing cases”, we were interested in examining whether the link between discrimination and cognition within this subgroup differed from those who identified a single or multiple reasons for discrimination. Thus, discrimination reason count was dummy coded as a multi-categorical moderator, with “no (zero) reasons” as the reference category (0 = reported discrimination but did not endorse reasons, 1 = one reason, 2 = multiple reasons). Finally, analyses examined the moderating effect of Black and White adults’ most commonly endorsed reason for discrimination (0 = did not endorse, 1 = endorsed) between discrimination frequency and each cognitive outcome.

Across all moderation analyses, participants’ gender, education (years), and number of health conditions were included as covariates.^1^ Continuous predictors were meancentered, and terms were estimated with 5,000 bootstrapped iterations to compute bias corrected 95% confidence intervals.

## Results

### Sample demographics related to ambulatory cognitive performance, discrimination frequency, and characteristics of perceived reasons for discrimination

Independent samples *t*-tests indicated that Black adults made more spatial working memory errors (*t*(253.57) = −6.05, *p* < 0.001), had poorer short-term memory binding performance (*t*(257.09) = 4.47, *p* < 0.001), and exhibited slower processing speeds (*t*(271) = −3.51, *p* < 0.001) compared to White adults.^2^ Black adults also reported more frequent perceived discrimination (*t*(271) = −4.96, *p* < 0.001) and a greater number of reasons perceived to underly discrimination (*t*(211.36) = −4.04, *p* < 0.001) than did White adults.

**Fig. 2:**
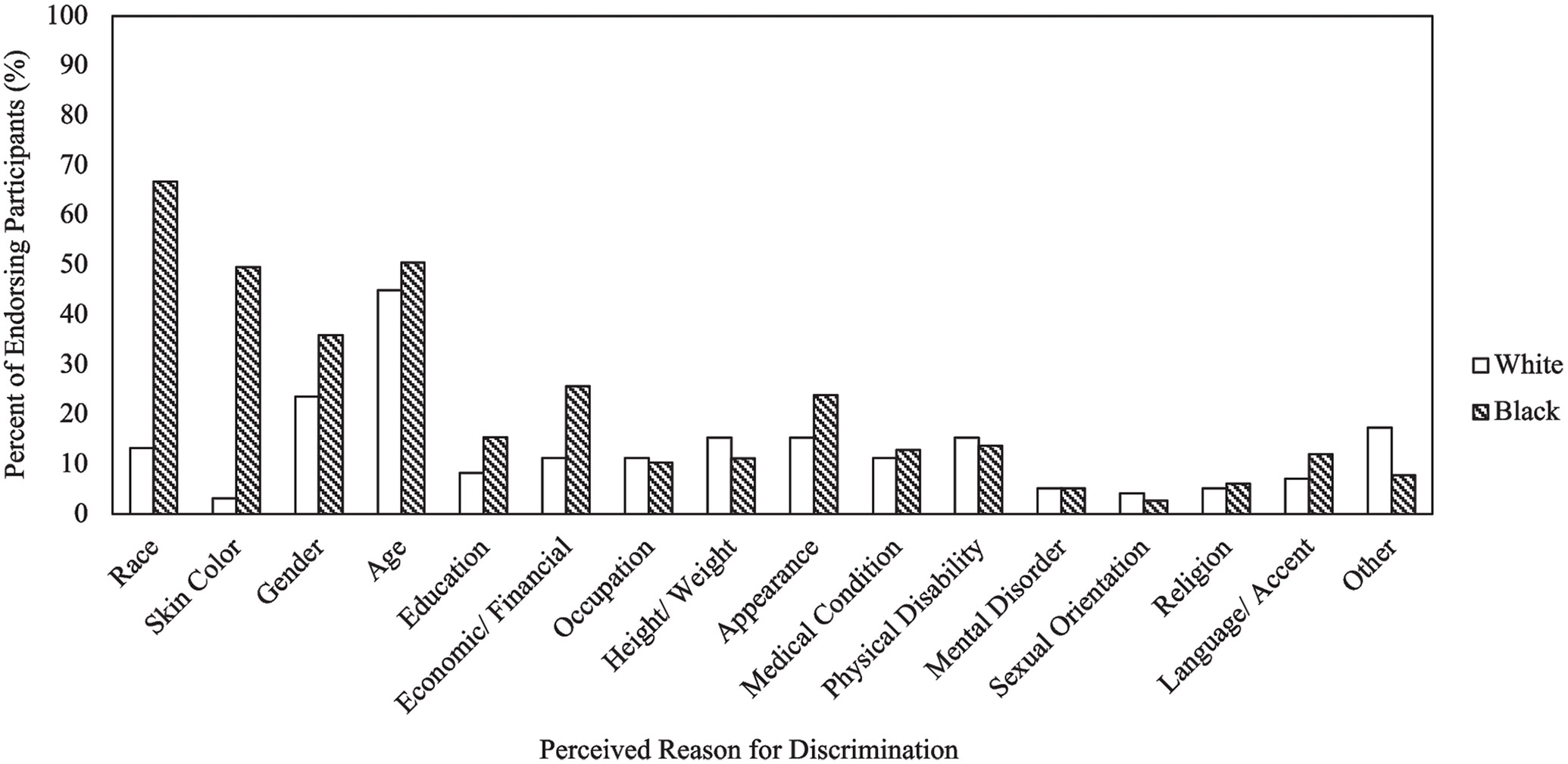
Percentage of Black and White adults who endorsed specific reasons as underlying their discriminatory experiences

We next examined the proportion of Black and White participants who reported experiencing some extent of discrimination and either endorsed zero, one, or multiple reasons as underlying their discriminatory experiences (i.e., reason count). Black and White adults differed on endorsed reason count (*χ^2^*(2) = 22.72, *p* < 0.001). Proportionally more Black (80.3%) than White (53.1%) adults endorsed multiple reasons for their discriminatory experiences, whereas more White (31.6%) than Black (7.7%) adults endorsed a single reason. A similar proportion of White (15.3%) and Black (12.0%) participants did not endorse any reasons for their discriminatory experiences. Regarding types of reasons (Fig. 2), Black participants most frequently endorsed their race (66.7%) whereas White participants most frequently endorsed their age (44.9%) as the reason for discrimination. More Black than White adults endorsed their race (*χ^2^*(1) = 62.30, *p* < 0.001) but the proportion of Black and White adults that endorsed their age did not differ (*χ^2^*(1) = 0.65, *p* = 0.419). Some participants (*n* = 25) indicated “other” reasons for their discriminatory experiences. Follow-up qualitative descriptions revealed that these discriminatory experiences were attributed to other individuals’ mood or demeanor (*n* = 9), their own demeanor (e.g., being quite/shy, *n* = 3), poor customer service interactions (*n* = 2), politics (*n* = 2), family (*n* = 2), other options captured by the measure (e.g., using a walker, auditory complaints, skin color, income; *n* = 5), or more ambiguous reasons (e.g., “general life occurrences”, “identity”; *n* = 2).

### Racially stratified analyses: Characteristics of perceived reasons for discrimination in relation to discrimination frequency and cognition

Racially stratified analyses (see below) examined whether characteristics of the perceived reasons for discrimination (*W*) moderated associations of discrimination frequency (*X*) with ambulatory cognitive performance. All model statistics from the following analyses can be found in Supplemental Tables 1-3.

#### Models examining Black adults

##### Discrimination reason count in relation to discrimination frequency and cognition

**Fig. 3:**
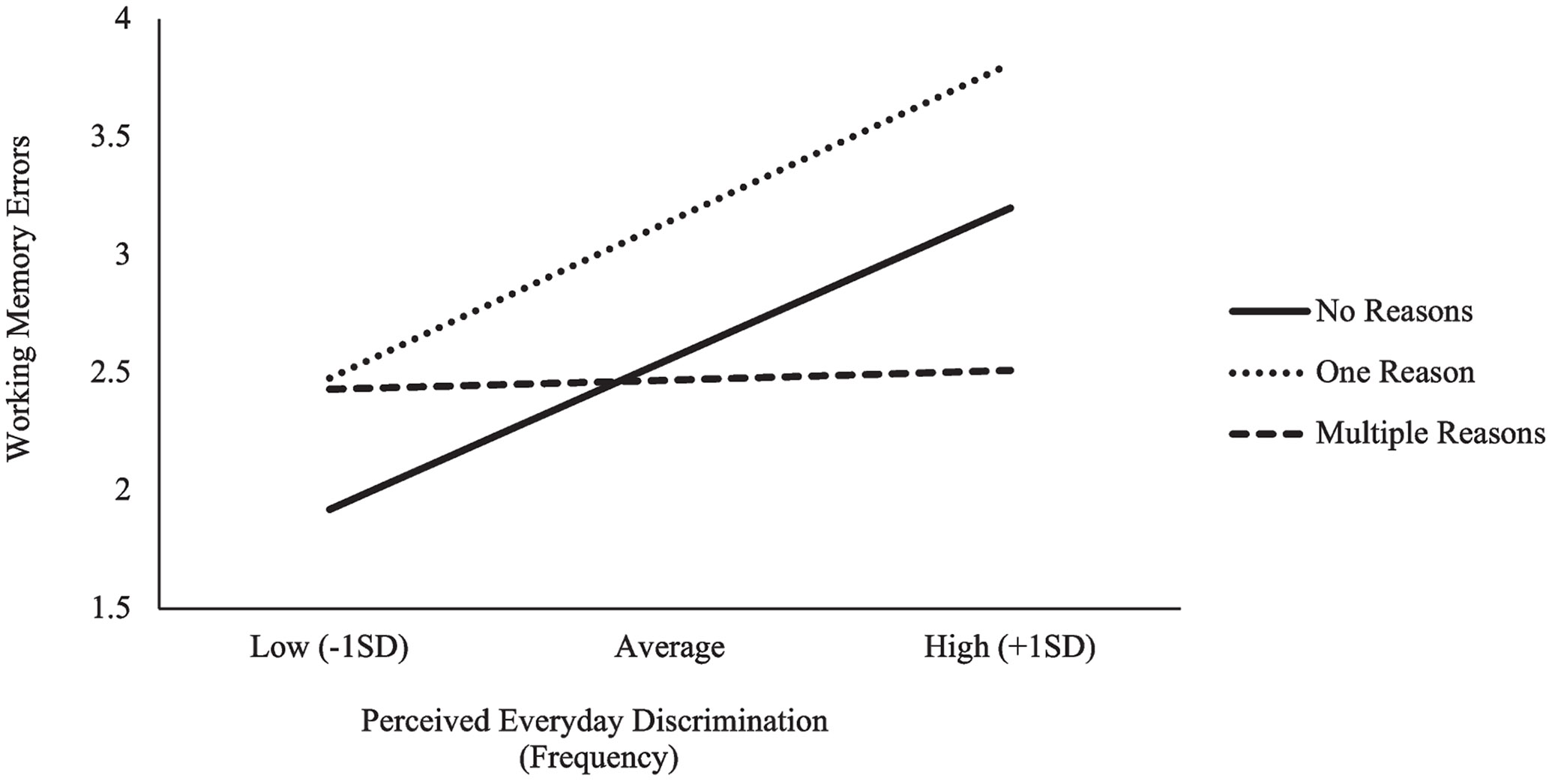
Interaction between perceived discrimination frequency, discrimination reason count, and spatial working memory errors among Black adults

When examining Black adults’ spatial working memory, a significant main effect of discrimination frequency was observed (*b* = 0.18, *p* = 0.003) but was subsumed by significant interaction effects; examination of the conditional effects (Fig. 3) revealed that discrimination frequency was associated with worse spatial working memory performance only among Black adults who attributed their perceived discrimination to one reason (*b* = 0.19, *p* = 0.036) or who did not endorse any reasons (*b* = 0.18, *p* = 0.003). Discrimination frequency was not related to spatial working memory among Black adults who endorsed multiple reasons (*b* = 0.01, *p* = 0.536). Significant interaction effects revealed that the simple slopes differed between Black adults who endorsed multiple reasons relative to those who did not endorse reasons (*b* = −0.17, *p* = 0.007) and marginally differed from those who endorsed one reason (*b* = −0.18, *p* = 0.053). The simple slopes between Black adults who endorsed one reason and those who did not endorse reasons did not significantly differ (*p* = 0.944).

Models that examined these same associations with Black adults’ short-term memory binding and processing speed did not reach statistical significance.

##### Race as a reason for discrimination in relation to discrimination frequency and cognition

**Fig. 4:**
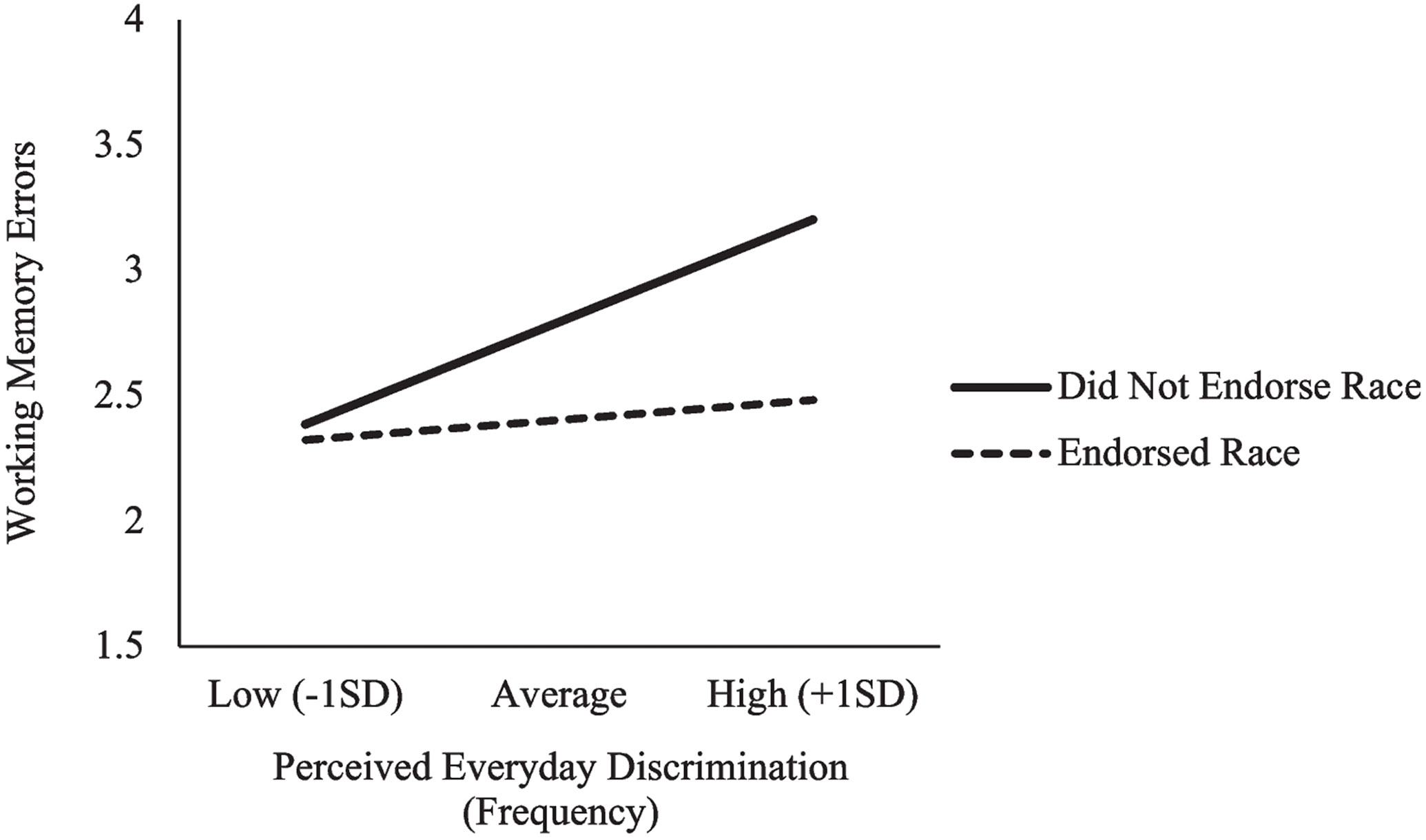
Interaction between perceived discrimination frequency, endorsement of race as a reason for discrimination, and spatial working memory errors among Black adults

Black adults most commonly endorsed race as a reason for discrimination; thus, moderation analyses assessed endorsement of racial discrimination in relation to discrimination frequency and spatial working memory among Black adults. A main effect of race as a reason was observed, such that Black adults who endorsed race as a reason made fewer spatial working memory errors than did individuals who did not endorse race (*b* = −0.39, *p* = 0.007). Additionally, race as a reason significantly moderated the relation between discrimination frequency and spatial working memory (*b* = −0.09, *p* = 0.043). Examination of the conditional effects (Fig. 4) indicated that more frequent discrimination was associated with more spatial working memory errors among Black adults who *did not* endorse race as a reason (*b* = 0.12, *p* = 0.005); this relationship was not significant for Black adults who endorsed race as a reason (*p* = 0.251). On an exploratory basis, we examined whether Black adults who endorsed or did not endorse race as a reason differed in terms of gender or education, as these covariates had been significant predictors in the tested model; however, the groups did not differ by gender (*p* = 0.759) or by education (*p* = 0.081). We also examined the moderating effect of racial discrimination in the relationships between discrimination frequency and short-term memory binding and processing speed, but none of the hypothesized pathways reached statistical significance.

#### Models examining White adults

##### Discrimination reason count in relation to discrimination frequency and cognition

Next, models examined whether White adults’ reason count moderated the link between discrimination frequency and cognition. The only significant relationship that emerged was in relation to short-term memory binding. Specifically, we found significant main effects related to the number of attributed reasons for discrimination. In comparison to White adults who did not endorse reasons, those who endorsed one reason had significantly better short-term memory binding (*b* = 0.17, *p* = 0.024) and those who endorsed multiple reasons had marginally better short-term memory binding performance (*b* = 0.13, *p* = 0.061). These findings did not vary by discrimination frequency and no other significant effects emerged. Hypothesized pathways in models examining White adults’ spatial working memory and processing speed did not reach statistical significance.

##### Age as a reason for discrimination in relation to discrimination frequency and cognition

White adults most frequently endorsed age as a reason for discrimination, and thus agebased discrimination was included as a moderator between discrimination frequency and ambulatory cognitive performance. However, no significant effects were observed on any of the cognitive measures.

## Discussion

The current study examined associations between discrimination and ambulatory cognitive performance in a sample of older Black and White adults. We expanded upon prior literature by exploring how discrimination frequency and characteristics of the perceived reasons for discrimination related to cognitive performance within daily life settings. Unlike past research, this study incorporated ambulatory cognitive assessments completed on mobile devices, offering the unique ability to assess the relationship between discrimination and cognitive functioning outside of traditional cognitive testing settings. In line with previous cross-racial examinations that assessed cognition in-lab (via neurocognitive tests; Babulal et al., 2019; Barnes et al., 2005), we found that Black adults performed worse on ambulatory tests of spatial working memory, short-term memory binding, and processing speed compared to White adults. Building from past research, the present study benefits from enhanced ecological validity, as we were able to assess performance within the environment that older individuals would typically utilize their abilities, and therefore provides novel insight into cognition within daily life. Consistent with past work (e.g., Ayalon & Gum, 2011), we also observed that Black adults reported experiencing discrimination more frequently and perceived a greater number of reasons to underly these experiences than did White adults. Findings related to racially stratified models that examined links between discrimination frequency and cognitive performance in relation to characteristics of the perceived reasons for discrimination are explored further in the sections that follow.

### Characteristics of Black adults’ discriminatory experiences and cognition

One important question was whether the perceived reasons for discrimination altered the associations between discrimination frequency and cognitive performance. Our analyses examined these characteristics separately within older Black and White adults who experienced discrimination. Interestingly, spatial working memory was the one cognitive outcome that uniquely related to discrimination in Black adults; reporting more frequent discrimination was associated with more spatial working memory errors. This finding is consistent with previous work (e.g., Barnes et al., 2012; Shankar & Hinds, 2017; Zahodne et al., 2020) and provides further support of the need for within-racial group investigations rather than relying on cross-racial comparisons.

Interestingly, the observed association between more frequent discrimination and worse spatial working memory was apparent only in Black adults who failed to identify reasons or identified a single reason for their discriminatory experiences. Black adults who identified multiple reasons for their discrimination exhibited no such association. There are several possible explanations for this finding. Endorsing multiple reasons for discrimination may indicate a more rapid appraisal and implementation of useful strategies that, in turn, might minimize distress associated with discrimination (for related work, see Nussbaum & Steele, 2007; Williams et al., 2019). Hence, Black adults who endorsed multiple reasons may have access to resources or strategies (e.g., social support, active coping skills) that buffer the negative impact of discrimination on cognition. In contrast, Black adults who did not identify reasons for discrimination or who attributed discrimination to one specific reason may not have access to, or may not capitalize on, available resources or strategies that could minimize distress and protect their working memory.

This finding observed for Black adults highlights the potential benefit of being able to label and/or comfortably express discriminatory experiences. From a clinical perspective, self-labeling diagnoses or traumatic experiences can help individuals understand their experiences and often relates to help-seeking behaviors (e.g., Horsfield et al., 2020; Oexle et al., 2020). Within the present context, being able to label reasons underlying discrimination may help individuals respond to these encounters in more positive ways (i.e., capitalizing on resources, avoiding internalizing these experiences) and ultimately, preserve their everyday cognitive performance. Conversely, individuals who perceive discrimination but do not label (or only assign one label) to the source of these experiences may not be seeking help to cope with this event.

Another possibly related explanation for the present findings is stereotype internalization. It has been shown that individuals who internalize (i.e., readily believe stereotypes to be accurate) are more likely to experience anxiety (Sosoo et al., 2020), lower self-esteem (Marquet et al., 2018), and poorer memory performance (Weiss, 2018) when they encounter discrimination. Within the current study, individuals who identified fewer reasons for discrimination may have been more likely to internalize these experiences than those who identified multiple reasons, ultimately impacting their cognition. If these same individuals are not seeking help or utilizing resources that can help them to actively cope with stressful situations, then there may be long term consequences for daily cognitive performance. Given that this pattern of findings was not anticipated, further examination of characteristics of discriminatory experiences in association with access to resources, help-seeking behaviors, and stereotype internalization may provide valuable insight into potential risks to cognition among Black adults.

When identifying reasons for discrimination, Black adults most frequently attributed their discriminatory experiences to their race. Notably, perceived racial discrimination moderated the relationship between discrimination frequency and spatial working memory; however, it was only in Black adults who did *not* endorse race as a reason for discrimination that we saw more frequent discrimination associated with poorer spatial working memory. Although one might expect to see worse working memory performance among those who experienced racial discrimination (e.g., Keating et al., 2021), findings in this area are mixed. Our findings are consistent with another study which found that Black adults who attributed discrimination to race exhibited better cognitive health in association with more frequent discrimination (Sutin et al., 2015). Other related work has similarly found that Black adults who report greater lifetime discrimination exhibit better cognitive performance (Meza et al., 2022). The current observations align with existing literature which suggests that there is an association between identification of racial discrimination among older Black adults and cognitive performance, now extending these findings to better spatial working memory within daily settings. Possibly this identification process has associations with psychosocial factors (e.g., social support, coping mechanisms) that may contribute to resiliency in the face of discrimination (see also, Amano et al., 2024).

Interestingly, discrimination was associated only with Black adults’ spatial working memory. In line with past suggestions (Keating et al., 2021), it is possible that discrimination reduces attentional capacity as one 1) attempts to stay vigilant for potential discriminatory sources, or 2) ruminates over recent events, thereby influencing Black adults’ working memory. This may particularly be the case if a person is unable to label the source underlying these experiences. Furthermore, these findings contribute to existing research on the domain specificity of discrimination on cognition (Zahodne et al., 2020), as we did not observe effects of discrimination on the other ambulatory cognitive abilities. The present findings suggest that spatial working memory may be a specific cognitive ability influenced by discrimination in daily life, whereas processing speed and short-term memory binding appear largely preserved. In general, working memory is critical for many necessary tasks of daily living (e.g., planning, decision making; Baddeley & Hitch, 1974), and it will thus be helpful for researchers, practitioners, and policy makers to be cognizant of potentially modifiable factors that can negatively impact various facets of this essential ability. Continued investigations into discrimination and cognitive performance in daily life will elucidate the effects of discrimination on cognition and health and inform the development of effective intervention strategies.

### Characteristics of White adults’ discriminatory experiences and cognition

We observed that White adults who endorsed one or more reasons for their perceived discriminatory experiences had better short-term memory binding performance than did those who did not endorse a reason. Similar to arguments made about Black adults’ perceived reasons for discrimination and cognition, it is possible that identifying at least one reason for discrimination enables White adults to implement active coping strategies that help them overcome these experiences (e.g., Czarnek et al., 2019; Kalenzaga et al., 2019) and ultimately support their memory, regardless of the frequency of these events. This may have clinical implications for White adults, as labeling the sources of one’s experiences may improve help-seeking (Horsfield et al., 2020; Oexle et al., 2020) and benefit older White adults’ everyday short-term memory. Importantly, however, White adults’ cognition was not associated with discrimination frequency or the commonly endorsed reason for discrimination (i.e., ageism). These findings demonstrate the importance of examining discrimination *within* racial groups, as the frequency and characteristics of discriminatory experiences were differentially associated with Black and White adults’ cognitive performance.

It should be noted that White adults completed more years of education, on average, than did Black adults within the present study. Educational attainment has been suggested to buffer age-related cognitive declines (e.g., Lövdén et al., 2020), which may explain why we did not observe many associations among the present sample of older White adults. The benefits of high levels of education (e.g., col- lege/graduate education) on cognitive performance have been observed to be heightened for Black adults compared to White adults (Jean et al., 2019). Thus, it is possible that examining the same hypothesized links among Black adults with higher levels of education may yield different relationships that what was observed within the present study. Likewise, the inclusion of a larger sample of White adults with lower levels of education may also indicate different relationships between discrimination and ambulatory cognition. Considering risk for perceived discrimination and associated poor health outcomes have been observed in Whites from lower socioeconomic backgrounds (e.g., Bower et al., 2013), additional research with demographi- cally diverse older White adults may reveal whether effects related to demographic backgrounds extend to links between White adults’ discriminatory experiences and ambulatory cognition.

### Limitations and future directions

The present work offers insight into the relationship between discrimination and cognitive performance in daily life. However, we acknowledge several limitations. First, the cross-sectional design of the present work means that we were not able to determine directionality, such as whether discrimination influenced cognitive performance or if individuals’ cognitive abilities influenced their ability to recall discriminatory events and identify associated reasons. While the present work offers insight into links between older Black and White adults’ discriminatory experiences and cognitive performance within daily settings, it would be valuable in future research to obtain a larger sample of racially diverse older adults to address other individual differences that may contribute to the observed effects. As an example, the present study was underpowered to address gender differences in the hypothesized relationships, which may further highlight effects among individuals with intersectional identities (Meyer, 2003). It would, furthermore, be beneficial to address these relationships among a geographically diverse sample of older Black and White adults, as some of the observed relationships may differ. Relatedly, future research may also consider cultural perspectives (e.g., individualistic, collectivistic) that members of certain racial groups hold, as these may be possible mechanistic factors related to the perception and impact of discrimination (e.g., Hunter, 2008).

Although our measure of perceived discrimination is widely used and accepted, we were unable to determine why some participants who reported discrimination did not identify specific reasons for their experiences. It is possible that some participants did not follow instructions or were not aware of the second portion of the measure, and thus, did not identify reasons. Alternatively, unmeasured individual differences may have contributed to why participants were not able or unwilling to identify reasons. Notably, this measure was only administered once in the present study. Black adults’ prolonged and repeated exposure to systemic and interpersonal discrimination likely affects their cognitive well-being differently compared to White adults (e.g., Bleich et al., 2019). Experiences of discrimination likely vary within and across days; thus, the assessment in the present research may not have captured *everyday* experiences of discrimination.

Employment of EMA methods in future research should include daily assessments of perceived discrimination (see also Keating et al., 2021) in addition to daily cognitive performance. As our current data only included a single assessment of discrimination, we opted to aggregate cognitive performance across the two-week measurement period and treat it similarly to the discrimination items. Thus, we were unable to examine daily associations between perceived discrimination and changes in cognitive performance, as well as potential practice effects on the cognitive tests. Future EMA studies that link experiences of discrimination with objective cognitive performance on a daily or momentary level will help elucidate the causal direction of effects and to determine whether prolonged discrimination uniquely impacts Black adults’ risk for cognitive decline.

Although participants were demographically diverse and from heterogeneous backgrounds, further research is needed that encompasses a larger and nationally representative sample. It is important to note that Black individuals in this study were born before the Civil Rights movement in the United States. As such, they likely experienced discrimination early in their lives and for a formidable time. Hence, the results obtained in this study might not generalize to the social experiences of younger Black Americans. Future investigations into the frequency and characteristics of discrimination will illuminate potential age- and cohort- related differences regarding the impact of discrimination on everyday cognitive performance and risk for cognitive decline. Researchers examining these constructs across age groups should strongly consider the use of EMA to more directly document relationships between psychosocial stressors, like discrimination, and cognition in everyday settings (Sliwinski et al., 2018) to improve our understanding of these constructs in daily life.

## Conclusions

The current study represents a first step in understanding the relationship between discrimination frequency, perceived reasons for discrimination, and everyday cognitive performance. Overall, these findings support the notion that the associations between perceived discrimination and cognitive performance in daily settings occur in a heterogeneous fashion between races. In line with prior recommendations, we hope this work encourages future research to use a within-racial group approach, especially for topic areas where racial disparities are known to exist. This will help to disentangle how the unique psychosocial experiences of Black adults may influence cognitive functioning and risk for cognitive impairment. Additionally, researchers should continue to examine experiences of discrimination and cognitive performance within daily settings to improve ecological validity, and to gain a better understanding of the impact that such experiences can have on cognition in daily life. Such examinations may be particularly pertinent when conducted in older adults and within race.

## Supplementary Material

Harrington et al Supplemental Tables

**Supplementary Information** The online version contains supplementary material available at https://doi.org/10.1007/s12144-024-07266-w.

## Figures and Tables

**Table 1.** Sample demographics

	Black Adults (*n* = 134)			White Adults (*n* = 139)		
	*n*	Range	*M*(*SD*) or %	*n*	Range	*M*(*SD*) or %
Age		70 – 89	77.4 (5.0)		70 – 90	77.1 (4.8)
Sex/Gender						
Men	30		22.4%	61		43.9%
Women	104		77.6%	78		56.1%
Marital Status						
Married	31		23.1%	59		42.4%
Separated	4		3.0%	0		0.0%
Widowed	41		30.6%	30		21.6%
Divorced	38		28.4%	19		13.7%
Never Married	20		14.9%	31		22.3%
Income						
Less than $15,000	18		13.4%	7		5.0%
Between $15,001 & $30,000	52		38.8%	35		25.2%
Greater than $30,000	62		46.3%	94		67.6%
Refused	1		0.7%	2		1.4%
Don’t know	1		0.7%	1		0.7%
Education		5 – 21	14.1 (3.2)		5 – 25	16.2 (3.6)
Number of Health Conditions		0 – 7	2.5 (1.3)		0 – 6	2.2 (1.4)
Spatial Working Memory			2.5 (0.7)			1.9 (0.9)
Short-Term Memory Binding			0.6 (0.3)			0.7 (0.3)
Processing Speed (ms)			3,413.6 (919.5)			3,036.1 (856.9)
Perceived Everyday Discrimination			13.81 (4.0)			11.6 (3.5)
Number of Endorsed Reasons for Discrimination	117		3.49 (2.6)	98		2.11 (2.4)

Full sample *n* = 273; Number of Endorsed Reasons includes fewer participants because only participants that reported experiencing some extent of discrimination received follow-up questions on their perceived reasons for discrimination

## Data Availability

Data were from the Einstein Aging Study (EAS): study information, codebooks, and a form to request data access are available through the Einstein Aging Study website, https://einsteinagingstudy.com.

## Footnotes

1 It should be noted that separate sensitivity analyses were conducted that included age and depressive symptoms as covariates. The observed findings were not meaningfully changed in either of the sensitivity analyses.

2 Significant differences between Black and White adults’ performance on the ambulatory cognitive tests remained when controlling for gender and education (number of years).
