# Supplementary material for: Characteristics of discrimination and ambulatory cognitive performance among older Black and White adults
Source: Curr Psychol. Author manuscript; Available in PMC 2026 Mar 4. (PMC12956292; doi:10.1007/s12144-024-07266-w)
Supplement: Harrington et al Supplemental Tables [file NIHMS2139457-supplement-Harrington_et_al_Supplemental_Tables.pdf]

Characteristics of Discrimination and Ambulatory Cognitive Performance among Older Black and White Adults: Supplementary Materials

Supplemental Table 1

Models Examining Discrimination Reason Count as a moderator in the Link between Discrimination Frequency and Ambulatory Cognitive Performance Separately Among Black and White Adults

	Black Adults (n = 117)		White Adults (n = 98)	
Spatial Working Memory (Y)	b	SE	b	SE
Discrimination Frequency (X)	0.18**	0.06	-0.01	0.09
Endorsed One Reason (W_1)	0.58	0.35	-0.35	0.27
Endorsed Multiple Reasons (W_2)	-0.09	0.19	-0.23	0.25
X*W_1	0.01	0.11	-0.00	0.10
X*W_2	-0.17*	0.06	-0.00	0.10
C_1: Gender	0.53***	0.14	0.39*	0.17
C_2: Education	-0.05*	0.02	-0.09***	0.02
C_3: Number of Health Conditions	-0.03	0.04	0.13*	0.06
Constant	2.90***	0.33	3.20***	0.44
	R^2 = .215<br>F(8, 108) = 3.69, p < .001		R^2 = .245<br>F(8, 89) = 3.62, p = .001	

Short-Term Memory Binding (*Y*)	*b*	*SE*	*b*	*SE*
Discrimination Frequency (*X*)	-0.02	0.03	0.02	0.03
Endorsed One Reason (W_1)	-0.04	0.17	0.17*	0.07
Endorsed Multiple Reasons (W_2)	-0.01	0.09	0.13	0.07
$X*W_1$	-0.02	0.05	-0.01	0.03
$X*W_2$	0.01	0.03	-0.01	0.03
C_1: Gender	-0.06	0.07	-0.01	0.05
C_2: Education	0.03**	0.01	0.03***	0.01
C_3: Number of Health Conditions	-0.00	0.02	-0.02	0.02
Constant	0.18	0.16	0.16	0.12
	$R^2 = .115$ $F(8, 108) = 1.75, p = .095$		$R^2 = .260$ $F(8, 89) = 3.91, p < .001$	

Processing Speed (*Y*)	*b*	*SE*	*b*	*SE*
Discrimination Frequency (*X*)	96.40	86.05	-15.91	41.77
Endorsed One Reason (W_1)	-963.84	512.50	-1.50	252.01
Endorsed Multiple Reasons (W_2)	-194.42	277.55	-415.53*	202.46
$X*W_1$	-153.18	156.65	2.71	101.17
$X*W_2$	-86.51	89.97	60.39	61.26
C_1: Gender	-11.67	208.53	-141.68	171.72

C_2: Education	-35.32	28.16	-40.37	23.85
C_3: Number of Health Conditions	86.55	64.24	2.35	58.92
Constant	3932.12***	477.04	3937.44***	436.76
	$R^2 = .085$<br>$F(8, 108) = 1.25, p = .280$		$R^2 = .129$<br>$F(8, 89) = 1.64, p = .124$	

Note. *$p < .05$; **$p < .01$; ***$p < .001$. Race was entered as a dichotomous moderator (White = 0, Black = 1).

Supplemental Table 2

Models Examining Race as a Perceived Reason for Discrimination as a moderator in the Link between Discrimination Frequency and Ambulatory Cognitive Performance Among Black Adults

Spatial Working Memory (*Y*)	*b*	*SE*
Discrimination Frequency (*X*)	0.12**	0.04
Race as a Reason (*W*)	-0.39**	0.14
*X**W*	-0.09*	0.04
C_1: Gender	0.52***	0.14
C_2: Education	-0.04*	0.02
C_3: Number of Health Conditions	-0.01	0.04
Constant	3.00***	0.33
	$R^2 = .187$	
	$F(6, 110) = 4.21, p < .001$	

Short-Term Memory Binding (*Y*)	*b*	*SE*
Discrimination Frequency (*X*)	-0.02	0.02
Race as a Reason (*W*)	0.10	0.07
*X**W*	0.01	0.02
C_1: Gender	-0.06	0.07
C_2: Education	0.03**	0.01
C_3: Number of Health Conditions	-0.00	0.02

Constant	0.15	0.16
	$R^2 = .123$	
	$F(6,110) = 2.56, p = .023$	

Processing Speed (*Y*)	*b*	*SE*
Discrimination Frequency (*X*)	54.00	59.20
Race as a Reason (*W*)	-285.84	207.18
*X**W*	-24.48	66.08
C_1: Gender	-56.13	204.83
C_2: Education	-28.52	28.33
C_3: Number of Health Conditions	81.08	64.03
Constant	3861.10***	470.79
	$R^2 = .058$	
	$F(6, 110) = 1.12, p = .353$	

Note. *$p < .05$; **$p < .01$; ***$p < .001$. Analyses based on constrained sample of Black adults who endorsed experiencing discrimination ($n = 117$). Race as a Reason was entered as a dichotomous moderator (0 = did not endorse, 1 = endorsed).

Supplemental Table 3

Models Examining Age as a Perceived Reason for Discrimination as a moderator in the Link between Discrimination Frequency and Ambulatory Cognitive Performance Among White Adults

Spatial Working Memory (*Y*)	*b*	*SE*
Discrimination Frequency (*X*)	0.01	0.04
Age as a Reason (*W*)	-0.31	0.17
*X**W*	-0.04	0.06
C_1: Gender	0.43**	0.17
C_2: Education	-0.10***	0.02
C_3: Number of Health Conditions	0.12	0.06
Constant	3.17***	0.43
	$R^2 = .263$	
	$F(6, 91) = 5.40, p < .001$	

Short-Term Memory Binding (*Y*)	*b*	*SE*
Discrimination Frequency (*X*)	-0.00	0.01
Age as a Reason (*W*)	0.06	0.05
*X**W*	0.02	0.02
C_1: Gender	-0.01	0.05
C_2: Education	0.03***	0.01
C_3: Number of Health Conditions	-0.02	0.02

Constant	0.24	0.12
	$R^2 = .233$	
	$F(6, 91) = 4.59, p < .001$	

Processing Speed (*Y*)	*b*	*SE*
Discrimination Frequency (*X*)	48.50	37.06
Age as a Reason (*W*)	100.53	174.45
*X*W*	-44.83	57.94
C_1: Gender	-150.12	176.49
C_2: Education	-39.88	24.25
C_3: Number of Health Conditions	3.25	59.79
Constant	373.27***	445.05
	$R^2 = .072$	
	$F(6, 91) = 1.18, p = .326$	

Note. $^*p < .05$; $^{**}p < .01$; $^{***}p < .001$. Analyses based on constrained sample of White adults who endorsed experiencing discrimination (n = 98). Age as a Reason was entered as a dichotomous moderator (0 = did not endorse, 1 = endorsed).

Appendix

Abbreviated Perceived Everyday Discrimination Scale (William et al., 1997).

INSTRUCTIONS: Thinking of your day-to-day experiences, how often have any of the following things happened to you? For each one of these, would you say it occurs never, rarely, sometimes, or often?

1. Are you treated with less courtesy than other people?
2. Are you treated with less respect than other people?
3. Do you receive poorer service than other people at restaurants or stores?
4. Do people act as if they think you are not smart?
5. Do people act as if they think you are not as good as they are?
6. Do people act as if you are not capable of caring for yourself?
7. Are you called names or insulted?
8. Are you threatened or harassed?

What do you think is or are the main reasons for these experiences? Would you say your:

1. Race, ethnicity, ancestry, or national origins
2. Gender or sex
3. Age
4. Educational level
5. Economic or financial situation
6. Job or occupation
7. Height or weight
8. Shade of skin color

9. Some other aspect of your appearance
10. Medical condition
11. Other physical condition or disability
12. Mental health condition or disability
13. Sexual orientation
14. Religion
15. Language or accent
16. Other reason
